# Electrospun Core–Sheath Nanofibers with Variable Shell Thickness for Modifying Curcumin Release to Achieve a Better Antibacterial Performance

**DOI:** 10.3390/biom12081057

**Published:** 2022-07-29

**Authors:** Yubo Liu, Xiaohong Chen, Yuhang Gao, Yuyang Liu, Dengguang Yu, Ping Liu

**Affiliations:** 1 School of Materials and Chemistry, University of Shanghai for Science & Technology, Shanghai 200093, China; 191370146@st.usst.edu.cn (Y.L.); chengxiaohong_32@163.com (X.C.); 193742737@st.usst.edu.cn (Y.G.); 192432630@st.usst.edu.cn (Y.L.); ydg017@usst.edu.cn (D.Y.); 2 Shanghai Engineering Technology Research Center for High-Performance Medical Device Materials, Shanghai 200093, China

**Keywords:** core–sheath fibers, water-insoluble drug, variable sheath thickness, electrospinning, sustained release

## Abstract

The inefficient use of water-insoluble drugs is a major challenge in drug delivery systems. Core–sheath fibers with various shell thicknesses based on cellulose acetate (CA) were prepared by the modified triaxial electrospinning for the controlled and sustained release of the water-insoluble Chinese herbal active ingredient curcumin. The superficial morphology and internal structure of core–sheath fibers were optimized by increasing the flow rate of the middle working fluid. Although the prepared fibers were hydrophobic initially, the core–sheath structure endowed fibers with better water retention property than monolithic fibers. Core–sheath fibers had flatter sustained-release profiles than monolithic fibers, especially for thick shell layers, which had almost zero-order release for almost 60 h. The shell thickness and sustained release of drugs brought about a good antibacterial effect to materials. The control of flow rate during fiber preparation is directly related to the shell thickness of core–sheath fibers, and the shell thickness directly affects the controlled release of drugs. The fiber preparation strategy for the precise control of core–sheath structure in this work has remarkable potential for modifying water-insoluble drug release and improving its antibacterial performance.

## 1. Introduction

On-demand drug release has sparked remarkable attention in recent years, especially for water-insoluble drugs [1,2,3,4]. The original hydrophobic property of water-insoluble drugs remarkably limits their treatment efficiency [5]. Fortunately, diverse drug delivery systems have been intensively studied to address the issue of drugs with poor bioavailability, such as lipid nanoparticles [6,7], nanoemulgel [8,9], electrospun fibers [10], 3D printing [11], and hydrogel [12,13,14]. The water solubility and stability of drugs can be enhanced through controlled release or targeted drug delivery, thus promoting the absorption of drugs.

Electrospinning has been widely used to prepare pharmaceutical dosage forms [15]. A rapid fiber preparation process of polymers under high-voltage electric field demonstrates enormous promise in improving the loading efficiency of water-insoluble drugs. Traditional single-fluid electrospinning is a commonly used way of loading drugs that brings about short-term effective treatment to patients. With the development and exploration of electrospinning technology, the concept of multifluid electrospinning has gradually been proposed [16,17,18]. Multifluid refers to at least two fluids used to produce fibers during electrospinning, and coaxial and triaxial electrospinning technologies are the representative technologies in multifluid electrospinning [19,20,21,22]. In traditional coaxial electrospinning, sheath working fluids should be spinnable to supply a successful core–sheath nanostructure. After Yu’s group proposed an implementation strategy with an unspinnable working fluid to smoothen the fiber preparation (such as lipid, drug solution, and pure solvent), the modified coaxial electrospinning technology has rapidly developed [23]. Modified triaxial electrospinning is an evolution of modified coaxial electrospinning. Unspinnable fluids can manipulate the evaporation rate of the core solvent when they appear in the outer layer to produce fibers effectively [24]. Innovative strategies can be proposed on the basis of this idea to fabricate advanced functional materials. In modified triaxial electrospinning, the unspinnable working fluid optimizes the preparation of high-quality nanofiber, and the other two working fluids provide other potential storage places for drugs and polymers.

**Figure 1:**
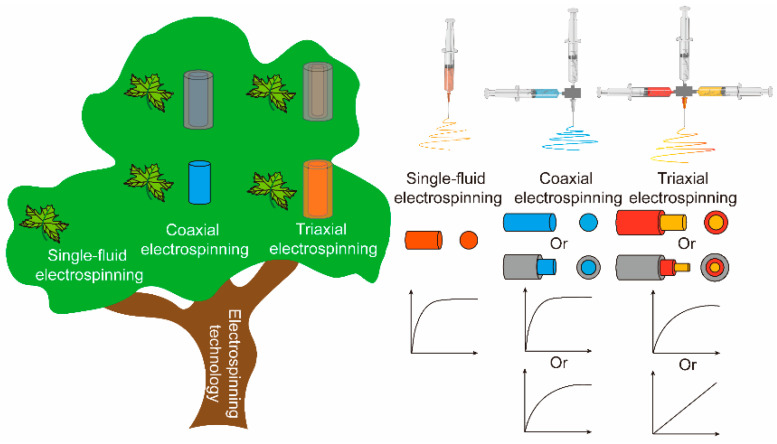
Schematic of electrospinning technology and its resulting fibers with different drug release performances.

Nanostructures have been a critical element for researchers to explore and develop dosage forms with tunable drug release [25,26]. The construction of multilayer pharmaceutical dosage form can provide a protective barrier to drugs. Advanced electrospinning technology can generate diverse fibrous structures and provide controlled drug release for water-insoluble drugs [27]. As shown in Figure 1, typical coaxial or triaxial electrospinning is used to prepare multilayer drug-loading fibers. In coaxial electrospinning, the sheath working fluid determines the monolithic or coaxial structure of the prepared fibers. Pure solvent solution without polymers has a positive effect on the preparation of high-quality monolithic fibers. Monolithic fibers have burst release in the early stage, whereas coaxial fibers delay the time of drug release [28,29,30]. Similarly, triaxial electrospinning can fabricate coaxial or triaxial fibers. Triaxial fibers have a better sustained-release profile than coaxial fibers. Considering that the drug-enriched core is theoretically blocked from drug release by two other protective layers, increasing the thickness of the sheath layer in core–sheath fibers can improve the sustained-release properties of drugs [31]. The preparation of coaxial structure is less difficult than that of triaxial structure.

Here, core–sheath nanofibers with variable sheath thickness were successfully fabricated by manipulating the flow rate of the middle fluid during modified triaxial electrospinning. Cellulose acetate (CA), a filament-forming polymer, has been explored as an ideal carrier for drugs in recent years and is used as sole matrix in this work. Curcumin, a low-cost Chinese herbal active ingredient with antibacterial properties, can play a remarkable bioactive ingredient role in the treatment of various diseases [32,33,34]. However, poor water solubility remarkably affects the therapeutic efficiency of drugs. Improving the biocompatibility of such poorly water-soluble drugs is of great significance for the treatment of diseases. Exploring how to improve the utilization of active ingredient of traditional Chinese medicine is beneficial to the development of pharmaceutics. In this work, curcumin was loaded into the core layer of CA fibers as a model drug to improve its bioavailability, and core–sheath drug carriers with variable sheath thickness were able to precisely manipulate the release behavior to improve the antibacterial properties of drugs. Moreover, monolithic CA drug-loading fibers were applied as a control group in this work to discuss the characterization and performance of core–sheath nanofibers with variable sheath thicknesses. The release mechanism of drugs and their effect on antibacterial performance were analyzed in detail.

## 2. Materials and Methods

### 2.1. Materials

White CA powders with 39.8 wt% acetyl group and 3.5 wt% hydroxyl group were provided by the Aladdin Biochemical Technology Co., Ltd. (Shanghai, China). The model drug curcumin and organic solvents (such as acetone, N,N-dimethylformamide (DMF), and ethanol) were purchased from Sinopharm Group Chemical Reagent Co., Ltd. (Shanghai, China). Phosphate-buffered saline (PBS) and Tween 80, the main release medium, were also supplied by Sinopharm Group Chemical Reagent Co., Ltd. (Shanghai, China). Magenta and methylene blue dyes were supplied by Tianjin Zhiyuan Chemical Reagent Co., Ltd. (Tianjin, China). All chemicals and reagents were used as received.

### 2.2. Electrospinning

In this work, the drug/polymer-based working fluid consisted of 0.72 g CA and 0.12 g curcumin dissolved in 6 mL mixture of ethanol, DMF, and acetone (1:1:4, *v*/*v*/*v*). The other polymer-based working fluid, as a blank middle layer in the modified triaxial electrospinning process, was prepared using 0.72 g CA dissolved in 6 mL mixture solvents. The two polymer-based working fluids were stirred at a constant temperature of 60 °C for 6 h to obtain transparent and clear solutions. In addition to the two polymer-based working fluids, the mixture solvents were used as the outer fluid. Details are presented in Table 1. Four nanofibers were produced using a single-fluid and modified triaxial electrospinning process. The temperature and relative humidity were 25 °C ± 5 °C and 40 ± 10%.

In this work, drug/polymer-based working fluid was used as an inner fluid, whereas blank CA and mixture solvent working fluids were designed as middle and outer fluids, respectively. All working fluids were loaded in plastic syringes and connected by flexible tubes and homemade spinneret. High voltage was supported by YFSP-T Yunfan Instrument Co., Ltd. (Tianjin, China) through a conductive clip. Then, the flow rate of the middle fluid was manipulated to fabricate T1–T3 nanofibers (Table 1). In addition, monolithic F1 nanofiber prepared by single-fluid electrospinning was used as a control group.

## 3. Characteristics

### 3.1. Surface Morphology and Internal Structure of Nanofibers

Nanofiber samples were plated with gold under a nitrogen vacuum environment to endow conductivity. Then, samples were observed using a field-emission environmental scanning electron microscopy (FESEM, Hillsboro, FL, USA) at 15.3 mm (working distance) and a high voltage of 30 kV. The average diameters of nanofiber samples were measured by the NIH ImageJ software (Bethesda, MD, USA). Fiber samples should be collected on the copper mesh prior to the observation of internal structure, and then the double-distilled water is used to increase the bonding force between them. Transmission electron microscopy (TEM, Hitachi, Japan) was used to explore the internal structure of dried nanofibers.

### 3.2. Physical Form and Chemical Compatibility

In the X-ray diffraction (XRD) test, samples were flattened and analyzed by the precise MiniFlex 600 (Rigaku, Japan) from 10° to 70° at a constant rate of 5° per min. The chemical compatibility between fiber components was evaluated by attenuated total reflection Flourier transformed infrared (ATR-FTIR) spectroscopy, which was conducted from 500 to 4000 cm^−1^ and scanned eight times.

### 3.3. Water Contact Angle (WCA) and Swelling Performance

Each fiber sample was cut into a 70 × 20 mm^2^ rectangle and fixed flatly on a glass slide by using double-sided tape. The JC2000C1 interface tension measuring instrument (Shanghai, China) was conducted to test the WCA of samples at least six times and then recorded as mean ± SD.

About 10 mg sample was added with 500 mL PBS (pH = 7.2, 37.5 °C), and the mixture was shaken by a SHA-AB shaker (Changzhou, Jiangsu, China) at 50 rpm. The mass of fiber was accurately weighed at indicated time points and placed in a dry beaker (after tare deduction) three times, and data were presented as mean ± SD. The swelling efficiency ($E_{S}$) was evaluated by water uptake by using the following formula (Equation (1)):

(1) $$E_{S}=\left(M_{W}-M_{D}\right)/M_{D}$$

where $M_{W}$ and $M_{D}$ are the masses of fibers after (wet) and before (dry) water soaking, respectively.

### 3.4. Drug Release

Drug release was examined following the pulp method in the 2015 Chinese Pharmacopoeia. About 0.02 g fibers were accurately weighed and added with 200 mL PBS (pH = 7.2, 37.5 °C) containing 0.5% (*v*/*v*) Tween 80. The mixture was shaken at 50 rpm. About 4 mL solution was collected periodically and added with an equal amount of PBS containing Tween 80. UV–VIS spectrophotometry (Shanghai, China) was conducted to obtain the absorption values at 425 nm, and the cumulative release of drug was calculated on the basis of the standard curve (*y* = 0.1288*x* − 0.0715, *R* = 0.9994). Results were presented as cumulative release or relative release *C* in Equation (2) [35]. Drug-loading efficiency (*D*) was calculated by Equation (3) [36].

(2) $$C=\left(\rho_{n}\times 200+\Sigma_{i=1}^{n-1}\rho_{i}\times 4\right)/C_{0}$$

(3) $$D=C_{0}/T_{theoretical}$$

where $C_{0}$ is the total actual drug amount in fibers, $\rho_{n}$ is the drug amount in *n*th solution, and $\rho_{i}$ is the drug amount in *i*th solution. In addition, $C_{0}$ could be obtained by the drug concentration in the release medium (200 mL) where 0.02 g fibers were completely dissolved. $T_{theoretical}$ is the theoretical drug amount in fibers.

Four mathematic models, such as first-order kinetic (F), Higuchi (H), Rigter–Peppas (Q), and zero-order kinetic (Z) model, were used to further process drug data [37,38,39,40].

(4) $$F=F_{0}\left(1-\exp\left(-kt\right)\right)$$

(5) $$H=H_{0}+kt^{1/2}$$

(6) $$Q=kt^{n}$$

(7) $$Z=Z_{0}+kt$$

where $F_{0}$, $H_{0}$, and $Z_{0}$ are the initial drug amounts; *k* is the kinetic constant; *n* is the drug diffusion coefficient; and *t* is the time required for drug release.

### 3.5. Antibacterial Activity

Antibacterial activity of the sample was quantified by using the turbidity measurement method [41]. Prior to the antibacterial test, fibers were cut into 1 × 1 cm^2^ squares and sterilized through 365 nm UV irradiation for 1 h. *Escherichia coli* (*E. coli*) CMCC(B) 44102 and *Staphylococcus aureus* (*S. aureus*) CMCC(B) 26003 were purchased from Beijing Preservation Biotechnology Co., Ltd. (Beijing, China), and used as reference strains. *E. coli* was cultured in Luria Bertani (LB) broth at 37 °C and shaking speed of 225 rpm, whereas *S. aureus* was cultured in tryptone soy agar (TSA). Fibers were placed into the bacterial liquid with the optical density (OD_600_) value of about 0.1 for 24 h in a waterproof incubator maintained at 37 °C (Shanghai, China). The photosensitizing property of curcumin was associated with antibacterial activity, and a 13 w LED was placed 10 cm above the bacterial liquid [42,43,44]. OD was a key parameter in evaluating the antibacterial activity of fibers, and each fiber was subjected to three independent tests. The antibacterial efficiency ($E_{A}$) was calculated using the following Equation (8):

(8) $$E_{A}=\left(V_{OD-PBS}-V_{OD-Fibers}\right)/V_{OD-PBS}$$

where $V_{OD-PBS}$ is the OD_600_ value of pure bacterial liquid after culturing for 24 h and $V_{OD-Fibers}$ is the OD_600_ value of fibers cultured with bacterial liquid for 24 h. Three parallel tests were performed, and results are reported as mean ± SD.

## 4. Results and Discussion

### 4.1. Modified Triaxial Electrospinning Process

**Figure 2:**
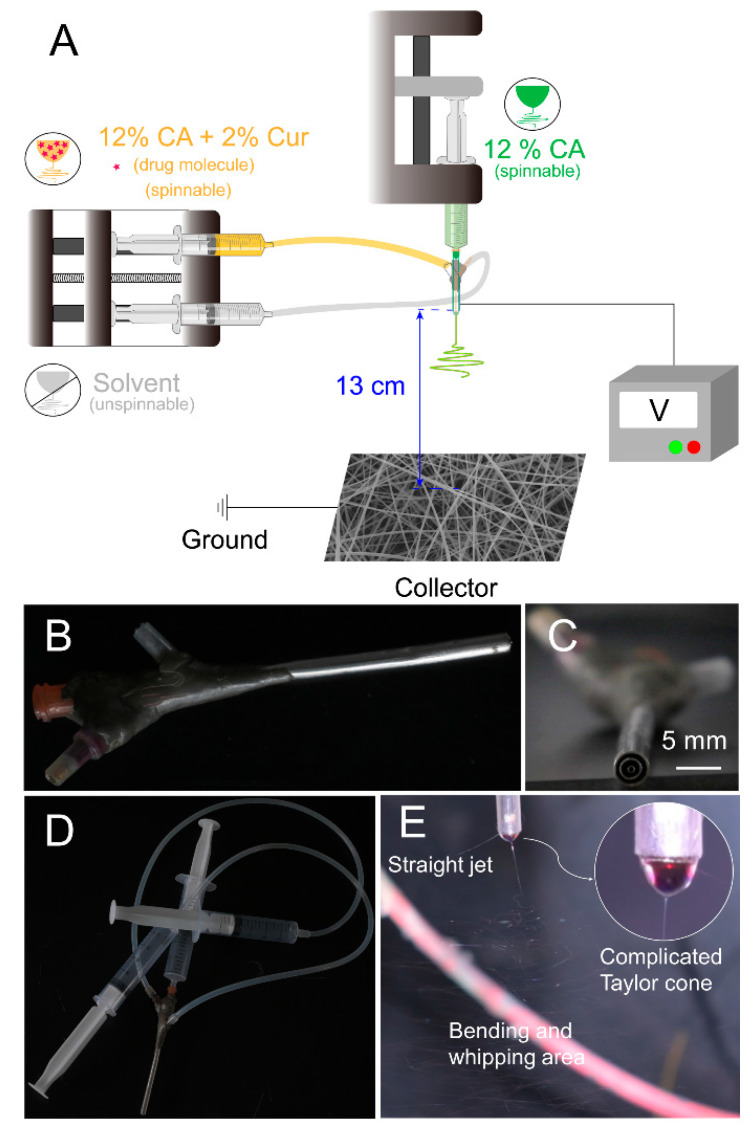
Scheme of modified triaxial electrospinning and its components. (**A**) Implementation of the modified triaxial electrospinning, consisting of pump, syringes, spinneret, aluminum foil collector, and power supply. (**B**) Homemade triaxial spinneret. (**C**) Partial enlargement of the head of the spinneret. (**D**) Connection between syringes and homemade triaxial spinneret by using transparent silicone tubes. (**E**) Modified triaxial electrospinning working process with a complicated Taylor cone (a clear triaxial structure) shown in the right corner.

In Figure 2A, modified triaxial electrospinning was implemented using unspinnable solvents and was different from the typical single-fluid or triaxial electrospinning. The spinneret is the key element for creating innovative functional fibers [45,46,47]. In this work, a homemade spinneret was conducted in the modified triaxial electrospinning process (Figure 2B). The metal capillaries 8 G, 12 G, and 20 G were the primary components that make up the homemade spinneret. As shown in Figure 2C,D, the spinneret was able to provide three different working fluids, consisting of three concentric circles and connected with plastic syringes via silicone tubes. A clear modified triaxial electrospinning process was observed by dying the working fluids. In this work, middle and inner working fluids were dyed magenta and methylene blue, respectively. In Figure 2E, a complicated Taylor cone exhibited clear color distribution, exhibiting blue–red–transparent from inside out. The evident hierarchical Taylor cone was the primary step for the structural fiber formation, giving a hint of the core–sheath structure in T1–T3 fibers.

### 4.2. Surface Morphology and Internal Structure of Nanofibers

**Figure 3:**
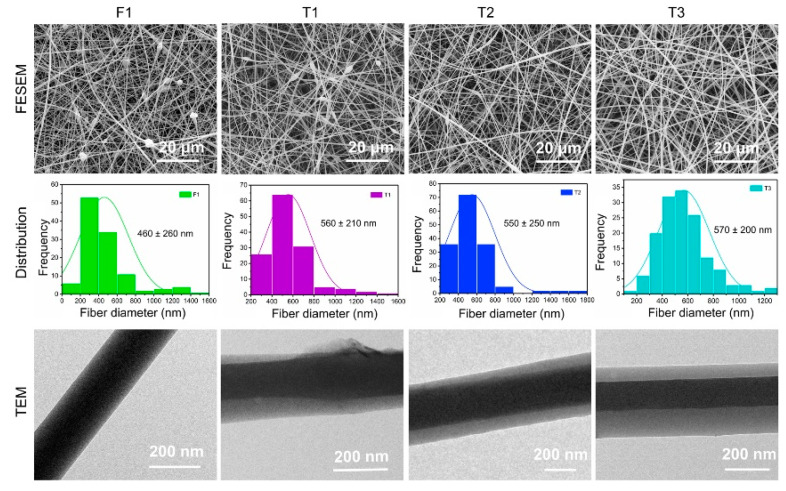
FESEM and TEM images of fiber products and statistical histogram of diameter.

As shown in Figure 3, the prepared fibers all had a linear morphology. In the recent literature, the modified electrospinning technology provides a positive effect on the morphology of fibers [48]. F1 fibers prepared by single-fluid electrospinning had a certain number of spindles, which was significantly improved in T1 fibers. In addition, a marked increase in the diameter of the two fibers (T1 > F1) was observed. This phenomenon could be contributed to the improvement and optimization of fiber morphology by the modified triaxial electrospinning technology and suggested the expanding effect of core–sheath structure on fiber diameter. In the modified triaxial electrospinning, increasing the pushing speed of spinnable middle working fluid had an enormous effect on fiber morphology, such as the spindles of T2 being less than those of T1, and T3 exhibited an excellent uniform cylindrical morphology. In addition, the increasing flow rate of the middle fluid had little effect on the average diameter of T1–T3 fibers.

The internal structure of fibers, such as monolithic structure of F1 and coaxial structure of T1–T3, is the most intuitive evidence for the successful preparation of the core–sheath structure and the effectiveness of modified triaxial electrospinning technique (Figure 3). Although fibers had a similar core–sheath structure, their internal structures (thickness of layers and location of the core layer) were different due to the different flow rate parameters. The core layer in the TEM image of T1 fibers was not strictly in the center of the sheath layer. In a typical coaxial or triaxial electrospinning technique, the flow rate of the outer fluid is always higher than that of the inner fluid to play a wrapping role. However, the flow rates of these fluids in this modified electrospinning process were the exact opposite, causing the inhomogeneous distribution of the core layer in the sheath layer (T1 and T2). This inhomogeneous distribution could be effectively improved when the flow rates reached the same (T3). Thickness details between layers are shown in Table 2.

### 4.3. XRD and ATR-FTIR Characteristics

**Figure 4:**
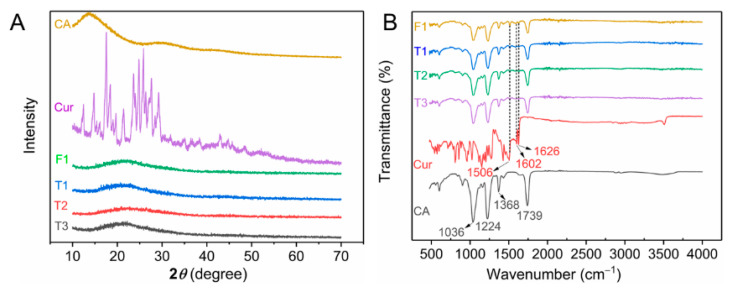
XRD pattern (**A**) and ATR–FTIR spectra (**B**) of raw materials (CA and curcumin powders) and prepared four fibers.

Figure 4A shows the XRD patterns of raw materials (CA and curcumin powders) and prepared fibers. Many sharp peaks indicated that curcumin had a typical crystal state, and several blunt halos or humps in the XRD pattern suggested that CA was amorphous [49]. Drug-loaded fibers, which were prepared by either single-fluid or modified triaxial electrospinning, lost any signal of drug crystals that exhibited smooth curves. Many studies demonstrated that electrospinning could convert the physical forms of drugs [50,51,52]. Before electrospinning, homogeneous and transparent working fluids indicated a highly uniform dispersion of drugs and polymers. The conversion of drugs from crystalline state to amorphous state was based on the rapid evaporation of solvents, which led drug molecules to be fixed instantly on the surface of solid fibers. Homogeneous fibers with an amorphous state of drugs could provide long-term storage unless the drug carrier was artificially damaged [53].

The ATR-FTIR spectra of raw materials and four prepared fibers are shown in Figure 4B. Several characteristic peaks appeared on the IR spectra of CA powders. The peaks at 1739, 1368, 1224, and 1036 cm^−1^ were attributed to the stretching vibration of the carbonyl (C=O) group, the stretching of methyl (-CH_3_) group, and ether (C-O-C) group [54,55,56]. In this work, CA was the primary polymer in all prepared fibers. Thus, these characteristic peaks were contained in the corresponding spectra of fibers (F1 and T1–T3). The IR spectra of curcumin had many characteristic peaks, whereas three signal peaks appeared in the IR spectra of drug-loaded fibers. The peak at 1506 cm^−1^ was due to the olefin (-C=C-) group, whereas peaks at 1602 and 1626 cm^−1^ were attributed to the carbonyl (-C=O) group [57,58,59]. These results suggested that curcumin was successfully encapsulated in fibers by the electrospinning process. No unexpected peak appeared in the IR spectra, indicating that curcumin had good compatibility with CA.

### 4.4. Performance of Functional Fibers

#### 4.4.1. Surface Wettability and Swelling Performance

**Figure 5:**
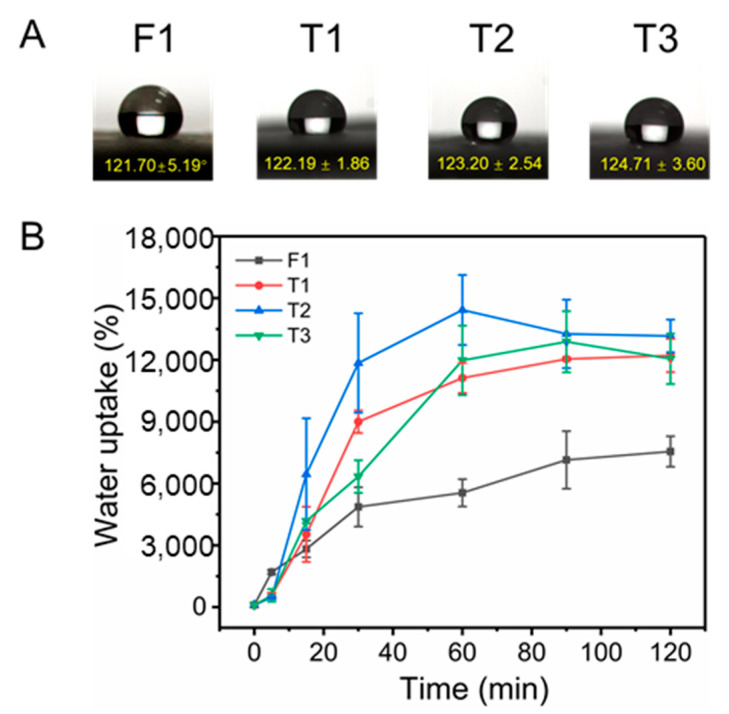
Water contact angle (**A**) and swelling efficiency (**B**) of the prepared fibers.

Surface wettability is a key index for the determination of the rate of drug release of drug carriers. The hydrophilicity of materials accelerates the release of drugs, whereas hydrophobicity has the opposite effect. In this work, WCA was used to evaluate the surface wettability of fibers. In Figure 5A, high WCAs (over 120°), which were higher than the critical value of 90°, suggested that all four fibers were hydrophobic. Results indicated that different electrospinning techniques did not influence the hydrophobicity of fibers when fibers were prepared with the same composition. The hydrophobicity of fibers (F1 and T1–T3) implied that drugs would be released at a slow rate.

Swelling performance of fibers is another index to evaluate the hydrophilic property, which is mainly used to evaluate the water storage capacity of fibers. Figure 5B exhibits the swelling performance of monolithic F1 and core–sheath T1–T3 fibers. CA, as a typical cellulose derivative, has excellent water uptake capacity [60,61,62]. The water uptake of F1 fibers reached 7555.38 ± 742.93% in only 2 h, whereas T1–T3 fabricated by modified triaxial electrospinning had almost double the water uptake of F1 fibers (T1, 12,213.47 ± 806.49%; T2, 13,161.35 ± 795.95%; T3, 12,054.76 ± 1226.01%). These results indicated that CA is a hydrophilic material, which was in contrast to the WCA results. The WCA test for only a few seconds could not allow sufficient intrusion of water molecules, thus leading to an artefact that CA is hydrophobic. The time of the swelling test is long enough for water molecules to invade, which shows the real hydrophilic performance of CA. In addition, the core–sheath structure could remarkably improve the water uptake ability of fibers, which was an important reflection of structure on performance. The flow rate of the middle working fluid in modified triaxial electrospinning had little effect on the swelling properties of fibers.

#### 4.4.2. Drug Release

The drug-loading efficiency was a prerequisite to evaluate whether the drug dosage forms could fully utilize drugs. In this study, the monolithic F1 fibers had a high drug-loading efficiency with 92.36 ± 5.53%, whereas core–sheath T1–T3 were 86.27 ± 2.30%, 80.16 ± 2.93%, and 78.81 ± 6.98%, respectively. Although a complicated structure might decrease the drug-loading efficiency to some extent, core–sheath fibers still had more than 75% drug-loading efficiency. In addition, drug dosage forms with high drug-loading efficiency need to be continuously administered so that drugs could be fully absorbed in the human body.

**Figure 6:**
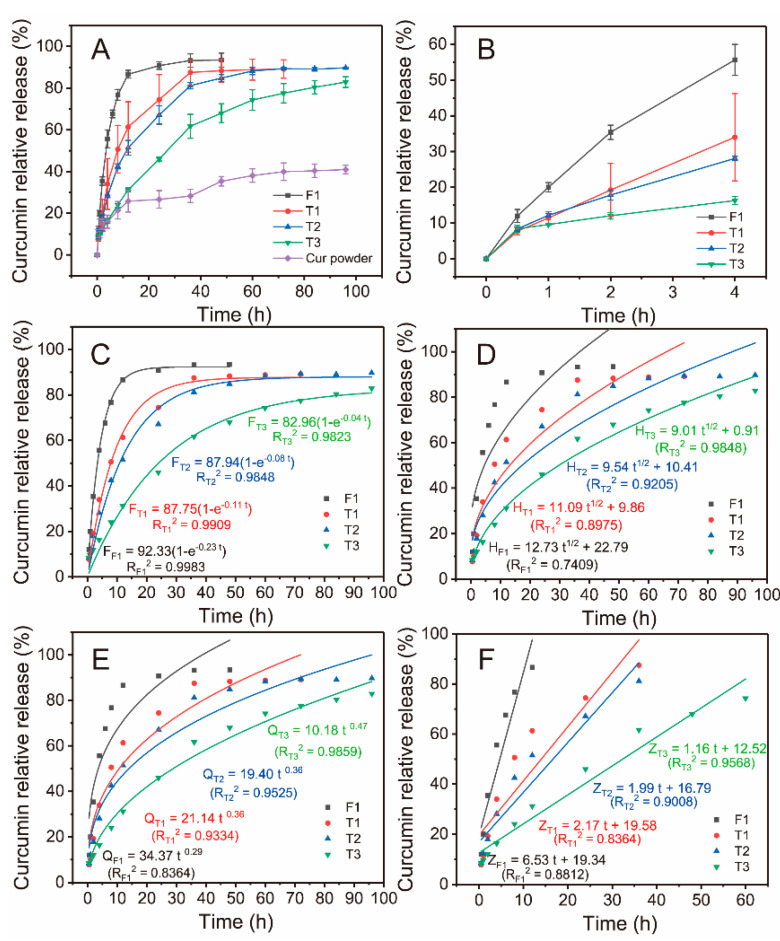
Drug release of prepared fibers. (**A**) Comparison of the relative release of curcumin from different fibers and pure curcumin powders. (**B**) Relative release of curcumin from fibers in the first 4 h. Fitting results of drug data according to the (**C**) first-order kinetic, (**D**) Higuchi, (**E**) Rigter–Peppas, and (**F**) zero-order kinetic models.

Figure 6A shows the effect of variable sheath thickness on drug release properties during modified triaxial electrospinning. Drug release in F1 reached saturation state after 24 h, whereas T1 had the same situation. However, the relative release of T1 was remarkably inhibited during the middle period. One special merit of drug-loaded core–sheath fibers was to protect drugs in the core layer by the sheath layer. Sheath thicknesses were controlled by the flow rate parameter, and the central location of the core layer was improved, as shown by TEM. An enlarged view of the drug release in the first 4 h is shown in Figure 6B. Core–sheath fibers effectively prevented the initial burst release of drugs. T3 provided a gentle early release of drugs. Sheath thickness had a positive effect on the sustained release of T1–T3 fibers, as shown in Figure 6A,B, and the profiles from 12 h to 36 h were almost linear. In particular, T3 had an almost linear profile for the first 36 h. The slow release rate of drugs in T3 fibers during the late stage provided remarkable potential for the treatment of chronic disease.

Several mathematic models were used to further process drug-release data. Figure 6C shows that all four fibers followed the first-order kinetic model due to the high correlation coefficient (*R*^2^), as shown in Figure 6C. In the Higuchi model, *R*^2^ > 0.9 indicated that drugs were dissolved through a diffusion process based on the porosity of polymers. Thus, only T2 and T3 were suitable for the Higuchi model, as shown in Figure 6D. The famous Rigter–Peppas model was used to analyze the dissolution pattern of drugs, as shown in Figure 6E. *n* ≤ 0.45 suggested that drug-loaded fibers had the typical Fickian diffusion, whereas 0.45 < *n* < 0.9 meant a hybrid mechanism of Fickian diffusion and skeleton erosion. All fibers had the Fickian diffusion mechanism. Variable sheath thickness caused by flow rate had little influence on the mechanism. Figure 6F exhibits the adaptability of the zero-order model for fabricated fibers; T2 and T3 had better fitness than other fibers (higher *R*^2^ > 0.9). T3 had a prominent and almost linear drug release profile of 60 h, which was caused by its thick sheath compared with T1 and T2. The zero-order release of drugs is an ideal performance for all dosages [63]. A slow-release rate and long-term dosing hold remarkable potential for chronic disease, which could reduce the dosing frequency [64]. The combination of structure and parameters substantially improves drug-release properties.

#### 4.4.3. Antibacterial Activity

**Figure 7:**
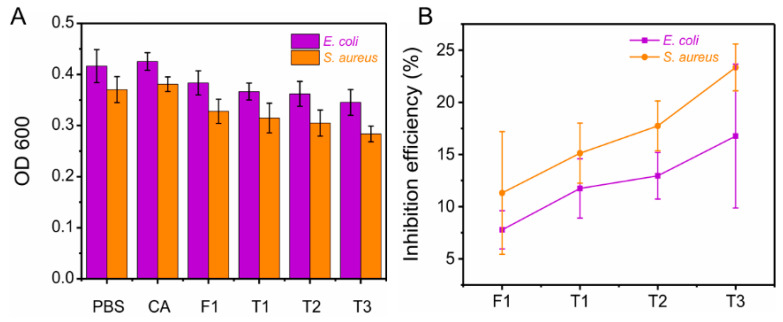
Antibacterial activity of four prepared fibers and control samples (PBS and blank CA fibers). (**A**) OD_600_ of bacterial liquid cultured with samples for 24 h. (**B**) Antibacterial efficiency of four prepared fibers.

Antibacterial activity of fibers is an important attribute for use in drug-carrier potential applications. Recent studies indicated that curcumin has a certain inhibition effect on Gram-positive (*S. aureus*) and Gram-negative (*E. coli*) bacteria [65,66,67]. Curcumin-loaded fibers had better anti-*S. aureus* properties than anti-*E. coli* properties, as shown in Figure 7A,B. For *S. aureus*, the inhibition efficiency of F1 (11.31 ± 5.88%) was weaker than those of core–sheath T1–T3 fibers (T1, 15.13 ± 2.88%; T2, 17.74 ± 2.39%; T3, 23.35 ± 2.24%). Core–sheath nanofibers with controllable sheath thickness could elaborately improve the antibacterial properties. The sustained release of drugs prevented the rapid reproduction of bacteria [68,69]. Similarly, F1 had 7.78 ± 1.83% inhibition efficiency for *E. coli*, whereas the inhibition efficiency of core–sheath T3 fibers was 16.77 ± 6.89%. Among the three core–sheath fibers, T3 had the best antibacterial performance due to its good sustained-release property (thickest sheath layer). Antibacterial efficiency could be calculated by the number of colonies in the nutrient agar plate. Blank control CA fibers had no antibacterial activity. Manipulating the sheath thickness of core–sheath T1–T3 nanofibers synergistically promoted sustained drug release and inhibition of colony growth. These results infer that structural fibers have a potential optimization effect on antibacterial performance.

#### 4.4.4. Performance and Mechanism Analysis

**Figure 8:**
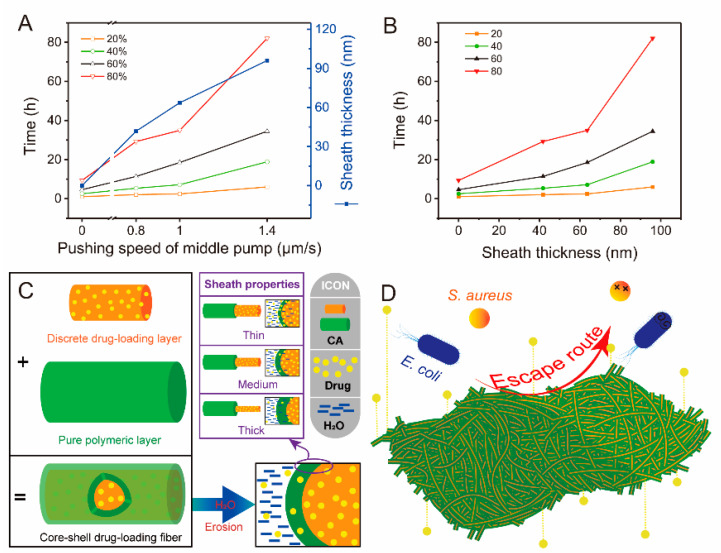
Relationship between the pushing speed of the middle pump (flow rate of the middle fluid), sheath thickness of core–sheath T1–T3 fibers, and release time of curcumin (**A**,**B**). Mechanism of drug release (**C**) and antibacterial activity (**D**) for manipulating the sheath thickness of core–sheath fibers.

The production process of materials has a positive effect on the optimization of material properties. In this work, the elaborate manipulation of parameters achieved the microscopic modulation of fiber structure at the nanoscale, which could improve the drug release performance. Figure 8A,B showed the relationship between flow rate of middle fluid, release time, and sheath thickness of core–sheath fibers. Sheath thickness (*s*) was manipulated by the flow rate of the middle fluid (*f*) and exhibited a strong linear relationship (*s* = 67.62*f* − 3.75, *R* = 0.9883). In addition, the flow rate is positively correlated with the time required for drug release (*t*). The regressed linear equations were *t*_20%_ = 3.15*f* + 0.35 (*R* = 0.8572), *t*_40%_ = 10.33*f* + 0.20 (*R* = 0.8455), *t*_60%_ = 19.90*f* + 1.31 (*R* = 0.9142), and *t*_80%_ = 46.89*f* + 1.37 (*R* = 0.8962), where *t_i_*_%_ is the time required for *i*% drug release. The results of drug release data (Figure 6A) indicated that shell thickness seems to have a direct effect on drug release performance, as shown in Figure 8B. Compared with Figure 8A, the sheath thickness had a similar linear relationship on release time of curcumin (*t*_20%_ = 0.05*s* + 0.41 (*R* = 0.9076), *t*_40%_ = 0.16*s* + 0.36 (*R* = 0.9017), *t*_60%_ = 0.31*s* + 1.83 (*R* = 0.9618), and *t*_80%_ = 0.72*s* + 2.80 (*R* = 0.9373)). The higher *R* in the above equations showed that the sheath thickness has a better effect on release time than flow rate.

Drug release in fibers is essentially a diffusion process by invaded water molecules. Two aspects dominated this process. First, whether water molecules could rapidly penetrate fibers depended on the hydrophilic properties of fibers. Good hydrophilic materials are beneficial for the intrusion of water and bring about quick drug release [70,71,72]. By contrast, hydrophobic materials provide a slow drug-release profile due to the difficulty of water penetration [73]. CA, the sole polymer used in this work, seemed like a hydrophobic material according to WCA results. However, the high water uptake performance after 120 min gave CA remarkable potential on sustained-release drugs. Thus, F1 fibers had a slow drug release profile (24 h) in the drug delivery systems due to the unique properties of CA. The other aspect was the storage space of drugs in fibers. The distance of drugs from the external environment of fibers indicates the time required for the journey of drug molecules [74]. The quick-release drugs in the early stage of F1 fibers originated from the surface of fibers or nearby. Drugs embedded in the center of fibers needed to travel long distances to reach the release medium, which led to a relatively flat drug-release stage. Thus, increasing the distance between drugs and release medium outside fibers could theoretically prolong drug release. Core–sheath fibers are common and effective materials for sustained-release drugs, in which the unique sheath layer could provide a barrier to prevent drugs from leaching the core [75,76,77]. Fortunately, the sheath thickness can be manipulated by adjusting the flow rate of the middle working fluid in modified triaxial electrospinning, as shown in Figure 8C. The thick sheath of core–sheath fibers indicated long diffusion distance for drug and water molecules, which determined the sustained drug delivery. Compared with the monolithic structure of F1, the thin or medium sheath thickness of core–sheath nanofibers increased the distance for drug journeys. T3, which had the thickest sheath of the three, had a good sustained-release profile (Figure 6A). This variable sheath thickness strategy provides on-demand drug release and good antibacterial property of materials (Figure 8D). Similarly, the combination of materials and structure in this work can inspire the production of new drug formulations.

## 5. Conclusions

To improve the utilization efficiency of the active ingredient of curcumin, we successfully prepared core–sheath nanofibers with controllable sheath thickness by using modified triaxial electrospinning to modify curcumin release. FESEM images showed that monolithic fibers had a few-spindle morphology, whereas core–sheath fibers showed a uniform linear morphology with increasing flow rate of middle working fluid during the modified triaxial electrospinning. The typical core–sheath fibers were proven by TEM, and sheath thickness increased as the flow rate of the middle fluid increased. XRD and FTIR tests indicated that curcumin was encapsulated in fibers with an amorphous form. Although all the prepared fibers were hydrophobic in the WCA test, they had good water uptake. Drug-release data showed that the thickness of core–sheath nanofibers provided a positive effect on drug release. The drug release from all prepared fibers strictly followed the Fickian diffusion at a certain rate, and core–sheath fibers with thick sheath had an almost linear zero-order release. The sustained-release process provided an efficient inhibition of *E. coli* and *S. aureus*. The variable sheath thickness strategy achieved a precise controllable release of curcumin and improved antibacterial properties of curcumin-loaded fibers. Sheath thickness precisely manipulated by flow rate provides a good sustained-release profile, and the good linear relationship between them is beneficial for the exploration of novel drug delivery systems.

## Figures and Tables

**Table 1:** Parameters of the four drug-loading electrospun nanofibers.

No.	Working Process	Working Fluid ^a^ (*w*/*v*)		The Pushing Speed of Pump (μm/s)			Structure
		Inner	Middle	Inner	Middle	Outer ^b^	
F1	Single-fluid electrospinning	12% CA + 2% Cur	-	1.4	-	-	Monolithic
T1	Modified triaxial electrospinning	12% CA + 2% Cur	12% CA	1.4	0. 8	1.4	Core–sheath
T2		12% CA + 2% Cur	12% CA	1.4	1.0	1.4	Core–sheath
T3		12% CA + 2% Cur	12% CA	1.4	1.4	1.4	Core–sheath

^a^ Applied voltage was approximately 10 kV during the electrospinning process, and the abbreviation Cur is curcumin. ^b^ Outer working fluid is the mixture of ethanol, DMF, and acetone (1:1:4, *v*/*v*/*v*), and the inner and middle working fluids were used the same mixture solvents to dissolve CA or Cur.

**Table 2:** Details of the thickness in modified triaxial electrospun nanofibers.

No.	Sheath Layer Thickness	Core Layer Thickness	Thickness Ratio of Core and Sheath Layer	Upper Sheath Thickness	Thickness Of Lower Sheath
T1	225 ± 16 nm	148 ± 9 nm	0.66	26 ± 5 nm	57 ± 23 nm
T2	382 ± 7 nm	257 ± 3 nm	0.67	48 ± 18 nm	79 ± 10 nm
T3	319 ± 3 nm	130 ± 0 nm	0.41	61 ± 4 nm	124 ± 2 nm

## Data Availability

All data presented in the study are included; further inquiries can be directed to the corresponding author.
